# Progressive multifocal leukoencephalopathy in an HIV patient was diagnosed by 3 times lumbar punctures and 2 times brain biopsies

**DOI:** 10.1007/s13365-020-00893-6

**Published:** 2020-08-24

**Authors:** Mengyan Wang, Zhongdong Zhang, Jinchuan Shi, Hong Liu, Binhai Zhang, Jun Yan

**Affiliations:** Xixi Hospital of Hangzhou, Hangzhou, 310023 China

**Keywords:** Progressive multifocal leukoencephalopathy, HIV, JC virus

## Abstract

Progressive multifocal leukoencephalopathy (PML) is a rare demyelinating disease of the central nervous system caused by JC virus (JCV) and is difficult to diagnose. We report on a male HIV-positive patient with PML finally diagnosed by 3 times lumbar punctures and 2 times brain biopsies. Negative results of JCV-PCR in cerebrospinal fluid (CSF) do not rule out the diagnosis of PML when clinical manifestations and neuroimaging features suspected PML. It is necessary to obtain new CSF and make repeat tests and even perform brain biopsy.

## Introduction

In HIV-positive individuals, central nervous system (CNS) infections remain a major cause of morbidity and mortality. And cerebral toxoplasmosis, progressive multifocal leukoencephalopathy (PML), tuberculous meningitis, cryptococcal meningitis, and cytomegalovirus infection are the common CNS opportunistic infections in HIV-positive individuals (Bowen et al. 2016). PML is a kind of demyelinating disease of the CNS caused by JC virus (JCV). In recent years, with the prevalence of AIDS and the widespread use of monoclonal antibodies, the incidence of PML has increased. Currently, HIV infection accounts for approximately 80% of new PML cases (Fournier et al. 2017). Herein, we report a case of PML detected by 3 times lumbar punctures and 2 times brain biopsies in an HIV-infected patient.

## Case presentation

A 47-year-old man with 15 years history of HIV infection had not received combined antiretroviral therapy (cART) before. One month ago, the patient started to have a skewed mouth with unclear speech, and after 2 weeks, the patient developed left limb movement disorder, gradually progressed to hemiplegia, and was referred to our hospital. Physical examination demonstrated that the patient’s left nasolabial sulcus became shallow, the corner of the mouth was skewed, the left limb muscle strength was 0, the muscle tone was normal, the sensation of shallow and deep was diminished, the left Babinski sign was positive manifested as hallux dorsiflexion, and the remaining four toes were fanned out.

**Fig. 1:**
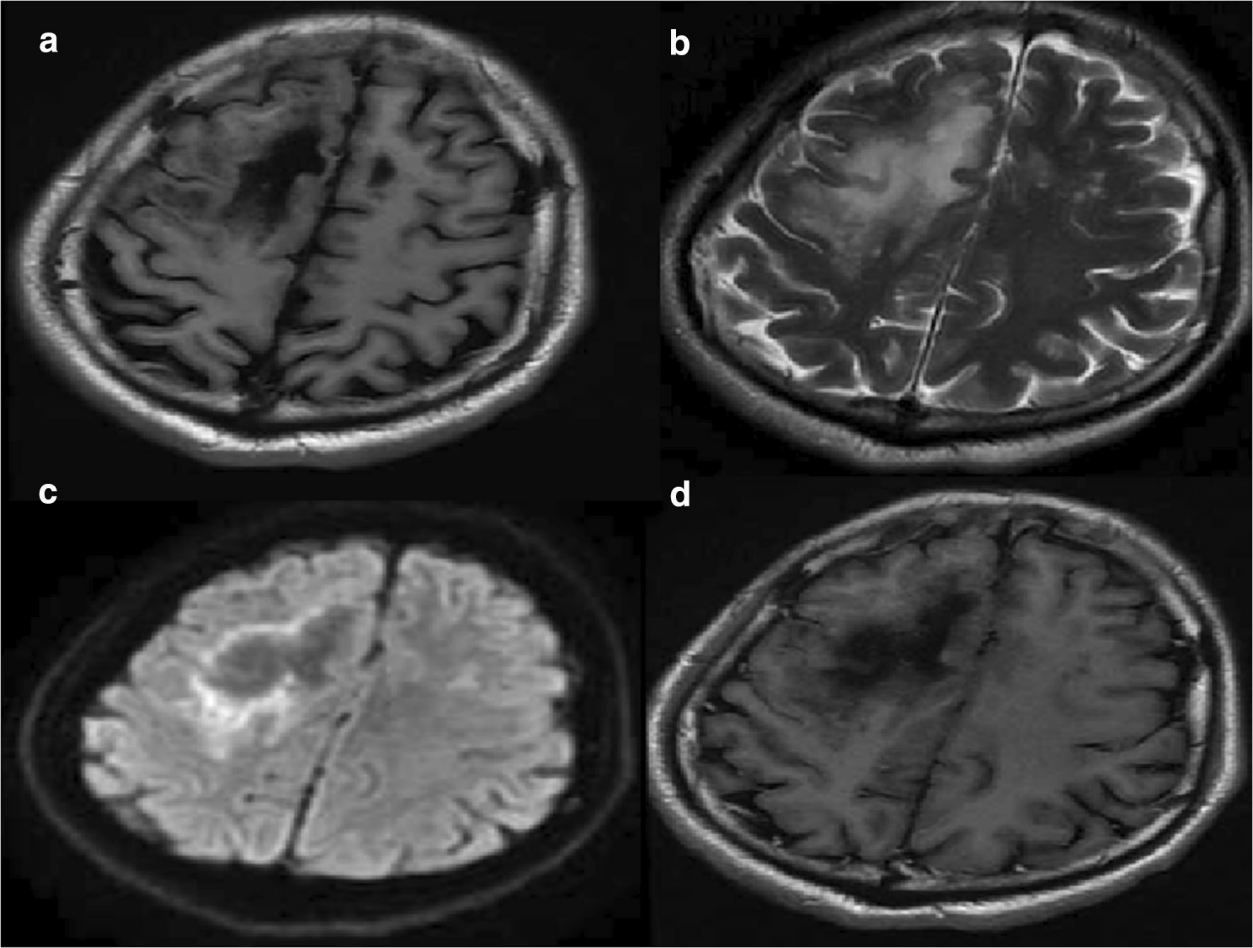
Magnetic resonance imaging results. T1-weighted images (**a**) showed patch-shaped signal shadows on the right frontal parietal occipital lobe and left frontal-temporal lobe. These lesions were hyperintense on T2-weighted images (**b**). Diffusion-weighted images (DWI) demonstrated ring-shaped marginal and small patch-like high signal shadows (**c**). No significant enhancement was seen after enhancement (**d**)

On blood examination, CD4 was 48/μL, and viral load was 11,800 IU/mL. There is no sign of bacterial, mycobacterium, and fungal infection. MRI of the brain showed massive necrosis of the right frontal parietal occipital lobe and left frontal-temporal lobe. Lacunar ischemia was scattered on both sides of the ventricle (Fig. 1). CSF analysis were within the normality range (glucose, chlorine), but the protein content was significantly increased to 1233 mg/L, and nucleated cells were significantly increased to 2*10^6/L. CSF cryptococcal antigen, treponema pallidum particle agglutination assay and culture were negative. India ink staining, acid-fast staining, and PCR technique for JC virus detection of CSF were also negative (Table 1). Pathological examination of brain biopsy showed that a small amount of brain tissue vascular congestion with erythrocyte exudation and edema of glial cell. Due to no evidences of any etiology, oral sulfamethoxazole-trimethoprim (sulfamethoxazole 1.2 g, trimethoprim 240 mg tid) was received to resist toxoplasmosis encephalopathy for 6 weeks and oral voriconazole 200 mg Q12H was received to resist intracranial fungal infections doubled on the first day for 3 weeks. The symptoms continue to worsen.

**Table 1:** Cerebrospinal fluid test results with 3 times lumbar puncture

Cerebrospinal fluid test	The first time	The second time	The third time
Glucose (2.5–4.4 mmol/L)	2.9	1.9	1.8
Chlorine (120–130 mmol/L)	122	120	119
Cell (0–10 10^6/L)	2*10^6/L	2*10^6/L	3*10^6/L
Protein (120–600 mg/L)	1233	1205	1336
PCR for JC virus detection	Negative	Negative	Positive

**Fig. 2:**
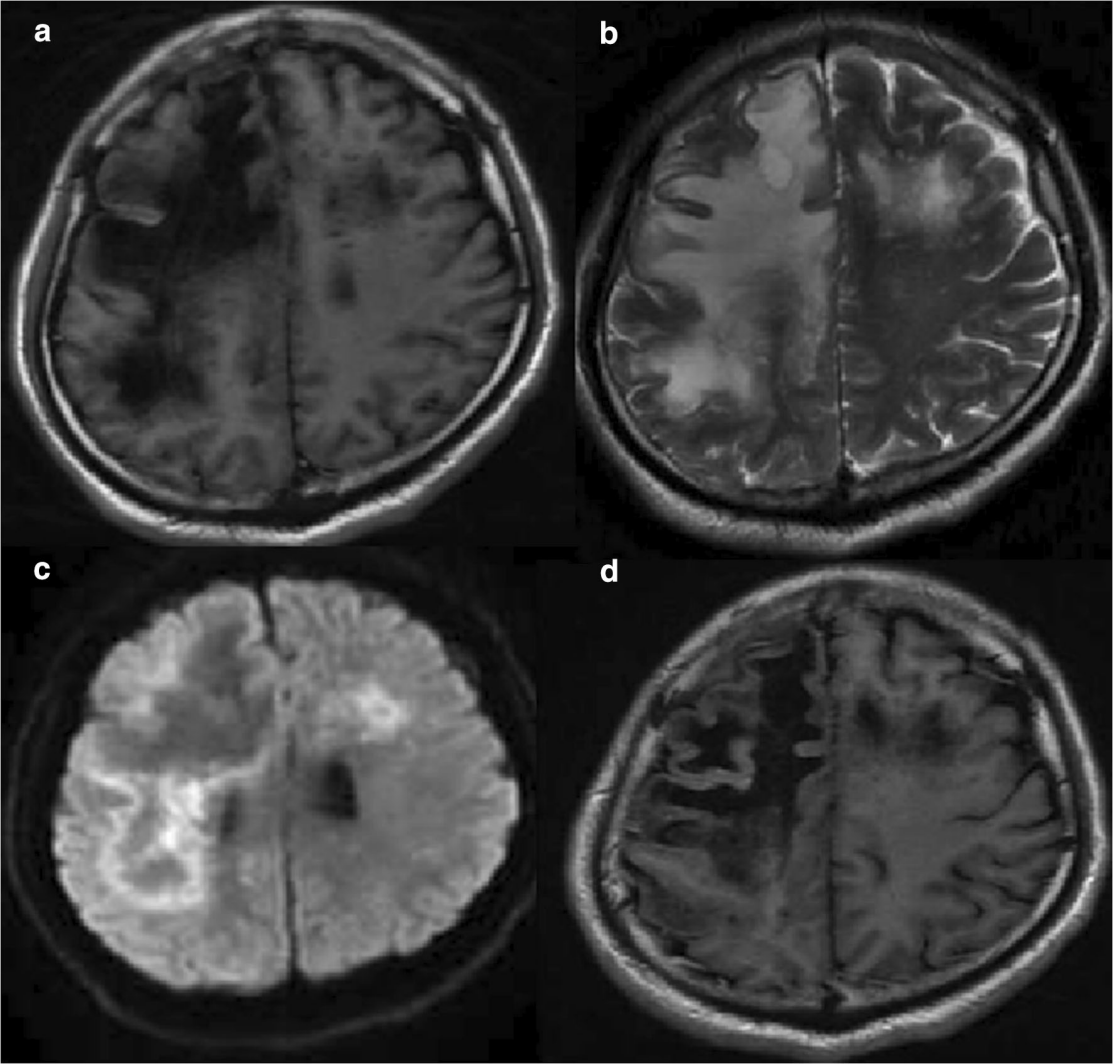
After 2 weeks of treatment, MRI of intracranial lesions got worse (**a**, **b**, **c**, **d**). The lesions were hypointense (dark) on T1 and hyperintense (white) in T2 sequences, showing no enhancement. DWI demonstrated ring-shaped marginal and small patch-like high signal shadows

**Fig. 3:**
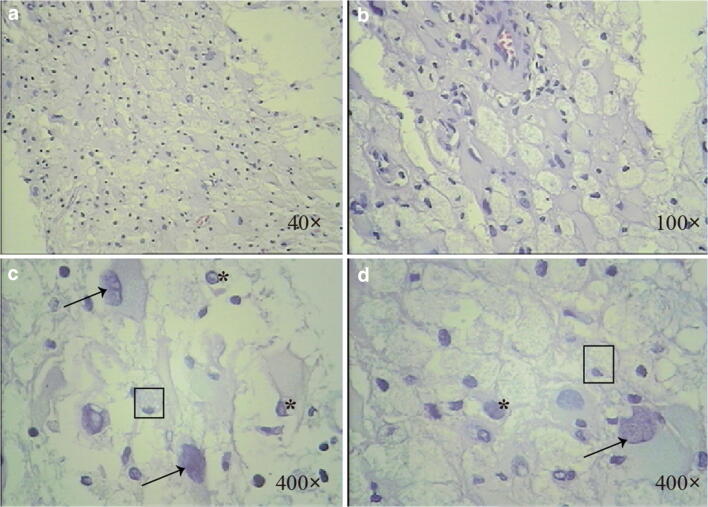
Hematoxylin and eosin (H&E) stained section showed atypical obese stellate collagen cell proliferation and a lot of foam cells. The glial nucleus was strangely shaped, and slightly eosinophilic intranuclear viral inclusions like nucleoli were seen. Interstitial small blood vessels were congested with peripheral lymphocyte infiltration. Arrow indicates infected astrocytes. Asterisk indicates infected oligodendrocytes. Square indicates histiocyte

After 2 weeks of treatment, MRI of the brain showed that intracranial injuries were more severe (Fig. 2). We repeated PCR with CSF for JC virus detection, and the result was still negative. We still had a suspicion of PML, and we did the third time lumbar puncture and the second time brain biopsy. Finally, we got the positive result of JC virus detection and the histopathology of brain biopsy conformed with PML (Fig. 3). After diagnosis of PML, we strengthened the cART (lamivudine + tenofovir + raltegravir + lopinavir/ritonavir). Due to progression of the disease, the patient died 2 months later, and the entire course was about 4 months.

## Discussion

PML is a kind of demyelinating disease of the CNS caused by the latent JC virus reactivating to infect oligodendrocytes and astrocytes. PML comprised up to 3–7% of HIV-1-infected patients before the advent of cART and made up to 18% of fatal CNS diseases (Cinque et al. 2009). It often occurs in HIV-infected patients with CD4 + cell counts < 200 cells/μL; unlike the other CNS-OIs, it can also develop in patients initiating cART with CD4+ cell counts above 200 cells/μL (Bowen et al. 2016; Falco et al. 2008).

The definitive diagnosis of PML was based on brain histopathology, and we increased the dependence on clinical features, imaging findings, and CSF-PCR for JCV due to the risk of brain biopsy (Berger et al. 2013). In HIV infection, PML could be confused with HIV encephalopathy, acute demyelinating encephalomyelitis, and primary CNS lymphoma through the imaging of white matter lesions without any evidence of etiology (Berger et al. 2013). In this case, the MRI of the patient showed CNS disease. In the exclusion of toxoplasmosis encephalopathy, HIV encephalopathy, and primary central nervous system lymphoma, we believed that the AIDS-related PML was the most likely. Therefore, it is urgent to get laboratory findings to help the diagnosis when clinical suspicion remains due to the ambiguity of noninvasive neuroimaging. PCR techniques of JCV in CSF have become the cornerstone of PML diagnosis. Its specificity for JCV is from 92 to 100% (McGuire et al. 1995; Weber et al. 1994), but the sensitivity was 89.5% in the pre-highly active antiretroviral therapy (HAART) era and in the HAART era was 57.5% (Marzocchetti et al. 2005). Sometimes there are false negative results, and the reasons may include the early phase of the disease (Zhai and Brew 2018), the technique of laboratory diagnosis, the low concentration and sample volume of the target DNA, and the loss of DNA during inspissation (Nakamichi et al. 2019).

Hence, negative PCR result of JCV does not rule out PML. In order to confirm the diagnosis, the PCR of JCV was repeated three times in this patient. Only the third time result was positive of JCV-DNA in CSF. Combined with the detection of nuclear virus inclusions in the second time brain biopsy, the diagnosis of PML in this case was clear. However, there is no effective treatment for PML. This patient was diagnosed with HIV-infected 15 years ago but had always refused HAART. In the case of HIV-associated PML, treatment with HAART can reduce mortality (Williamson and Berger 2017). However, the disease progressed rapidly, and the patient died after 2 months. The entire course of disease was about 4 months, which was consistent with literature reports.

Therefore, negative results of JCV-PCR in CSF do not rule out the diagnosis of PML when we strongly suspect PML by clinical manifestations and neuroimaging features. If the diagnosis of PML is still difficult to determine, we should obtain new cerebrospinal fluid and repeat tests and even perform brain biopsy. It remains the gold standard for diagnosis of PML.

## References

[CR1] Berger JR, Aksamit AJ, Clifford DB, Davis L, Koralnik IJ, Sejvar JJ, Bartt R, Major EO, Nath A (2013). PML diagnostic criteria: consensus statement from the AAN Neuroinfectious Disease Section. Neurology.

[CR2] Bowen LN, Smith B, Reich D, Quezado M, Nath A (2016). HIV-associated opportunistic CNS infections: pathophysiology, diagnosis and treatment. Nat Rev Neurol.

[CR3] Cinque P, Koralnik IJ, Gerevini S, Miro JM, Price RW (2009). Progressive multifocal leukoencephalopathy in HIV-1 infection. Lancet Infect Dis.

[CR4] Falco V, Olmo M, del Saz SV, Guelar A, Santos JR, Gutierrez M, Colomer D, Deig E, Mateo G, Montero M, Pedrol E, Podzamczer D, Domingo P, Llibre JM (2008). Influence of HAART on the clinical course of HIV-1-infected patients with progressive multifocal leukoencephalopathy: results of an observational multicenter study. J Acquir Immune Defic Syndr.

[CR5] Fournier A, Martin-Blondel G, Lechapt-Zalcman E, Dina J, Kazemi A, Verdon R, Mortier E, de La Blanchardiere A (2017). Immune reconstitution inflammatory syndrome unmasking or worsening AIDS-related progressive multifocal leukoencephalopathy: a literature review. Front Immunol.

[CR6] Marzocchetti A, Di Giambenedetto S, Cingolani A, Ammassari A, Cauda R, De Luca A (2005). Reduced rate of diagnostic positive detection of JC virus DNA in cerebrospinal fluid in cases of suspected progressive multifocal leukoencephalopathy in the era of potent antiretroviral therapy. J Clin Microbiol.

[CR7] McGuire D, Barhite S, Hollander H, Miles M (1995). JC virus DNA in cerebrospinal fluid of human immunodeficiency virus-infected patients: predictive value for progressive multifocal leukoencephalopathy. Ann Neurol.

[CR8] Nakamichi K, Kawamoto M, Ishii J, Saijo M (2019). Improving detection of JC virus by ultrafiltration of cerebrospinal fluid before polymerase chain reaction for the diagnosis of progressive multifocal leukoencephalopathy. BMC Neurol.

[CR9] Weber T, Turner RW, Frye S, Luke W, Kretzschmar HA, Luer W, Hunsmann G (1994). Progressive multifocal leukoencephalopathy diagnosed by amplification of JC virus-specific DNA from cerebrospinal fluid. AIDS.

[CR10] Williamson EML, Berger JR (2017). Diagnosis and treatment of progressive multifocal leukoencephalopathy associated with multiple sclerosis therapies. Neurotherapeutics.

[CR11] Zhai S, Brew BJ (2018). Progressive multifocal leukoencephalopathy. Handb Clin Neurol.

